# Value-modulated attentional capture is augmented by win-related sensory cues

**DOI:** 10.1177/17470218231160368

**Published:** 2023-03-23

**Authors:** Daniel Pearson, Meihui Piao, Mike E Le Pelley

**Affiliations:** 1 School of Psychology, UNSW Sydney, Sydney, NSW, Australia; 2 School of Psychology, The University of Sydney, Sydney, NSW, Australia

**Keywords:** Attention, reward, value modulated attentional capture, reinforcement learning

## Abstract

Attentional prioritisation of stimuli in the environment plays an important role in overt choice. Previous research shows that prioritisation is influenced by the magnitude of paired rewards, in that stimuli signalling high-value rewards are more likely to capture attention than stimuli signalling low-value rewards; and this attentional bias has been proposed to play a role in addictive and compulsive behaviours. A separate line of research has shown that win-related sensory cues can bias overt choices. However, the role that these cues play in attentional selection is yet to be investigated. Participants in this study completed a visual search task in which they responded to a target shape in order to earn reward. The colour of a distractor signalled the magnitude of reward and type of feedback on each trial. Participants were slower to respond to the target when the distractor signalled high reward compared to when the distractor signalled low reward, suggesting that the high-reward distractors had increased attentional priority. Critically, the magnitude of this reward-related attentional bias was further increased for a high-reward distractor with post-trial feedback accompanied by win-related sensory cues. Participants also demonstrated an overt choice preference for the distractor that was associated with win-related sensory cues. These findings demonstrate that stimuli paired with win-related sensory cues are prioritised by the attention system over stimuli with equivalent physical salience and learned value. This attentional prioritisation may have downstream implications for overt choices, especially in gambling contexts where win-related sensory cues are common.

Attention selects a limited amount of the information that we are presented with for further processing, and filters out the remaining information to be ignored. By shaping the information-gathering process, the way in which we direct our attention influences the choices that we make. For instance, choice alternatives that receive more attention are more likely to be chosen than less attended (but equally desirable) alternatives (Armel et al., 2008; Krajbich et al., 2010; Krajbich & Rangel, 2011; Shimojo et al., 2003). Moreover, manipulations that affect where attention is directed can bias choice (Gluth et al., 2018; Gwinn et al., 2019; Itthipuripat et al., 2015; Newell & Le Pelley, 2018), suggesting a causal role for attention in guiding decision-making. The overt choices that we make can therefore be thought of as the end point of an information processing cascade that begins with attentional selection (Pearson et al., 2022; see also Rangel et al., 2008). Thus, to fully understand why we come to make certain choices, we must understand why we come to attend to some choice alternatives over others.

There are a range of factors that influence how we allocate our attention (Awh et al., 2012; Failing & Theeuwes, 2018; Luck et al., 2021), and one of these is an item’s *learned value—*i.e., prior experience of a relationship between that item and reward. The influence of learned value on attention has been investigated using many different paradigms (for reviews, see Anderson, 2016a; Failing & Theeuwes, 2018; Pearson et al., 2022). In one example, Watson et al. (2019; see also Le Pelley et al., 2015) used a visual search task in which participants had to rapidly locate and respond to a diamond target that was presented among circles. Reward was earned according to response speed, with faster correct responses earning larger rewards. Errors resulted in loss of the reward that would have been earned for a correct response. On most trials, one of the circles (the *distractor*) was coloured, with all other shapes in grey. The colour of the distractor circle signalled the magnitude of reward that was available for a correct response to the target: a distractor in the high-value colour (e.g., orange) indicated that the current trial was a bonus trial in which reward would be multiplied by 10, a distractor in the low-value colour (e.g., blue) indicated that the current trial was not a bonus trial and so no multiplier would be applied. Therefore, across trials the orange distractor had high learned value and the blue distractor had low learned value. Importantly, even though the distractor’s colour signalled the magnitude of reward on each trial, it was never the target of search. As a result, the optimal approach to this task was to ignore the distractors entirely, since attending to them would slow search for the target and reduce reward. However, responses were found to be significantly slower (but no more accurate) on trials featuring a high-value distractor compared to trials featuring a low-value distractor. The implication is that the high-value distractor was more likely to be attended than the low-value distractor, even though this counterproductively resulted in participants earning reduced reward on high-value trials. This suggests that stimuli with relatively high learned value receive an automatic boost to their attentional priority, such that they involuntarily draw attention: a phenomenon known as *value-modulated attentional capture* (VMAC).

Recent findings suggest that VMAC may play a role in the development and/or maintenance of addiction and compulsive behaviour. Individuals with substance-use disorders (Field & Cox, 2008), and related problems such as disordered gambling, show attentional biases to addiction-related stimuli (for review, see Anselme & Robinson, 2020). These addiction-related attentional biases may represent a special case of the more general attentional bias to learned value (Anderson, 2016b, 2021). According to this account, the attentional priority of an addiction-related stimulus (e.g., flashing lights on a slot machine) is increased through pairing with reward (winning the “jackpot”), and thus these stimuli become more likely to automatically capture attention and subsequently influence behaviour (Pearson et al., 2022). In line with this idea, recent studies have shown that the magnitude of VMAC is associated with problematic substance use (Albertella et al., 2017; Albertella, Watson, et al., 2019; Anderson et al., 2013) and compulsivity-related problems including gambling (Albertella, Le Pelley, et al., 2019).

A separate line of research has demonstrated that the presentation of *win-related sensory cues—*i.e., audiovisual stimuli that accompany the delivery of reward—can alter the subjective experience of rewards and promote risky choice behaviour. For example, in the context of slot-machine gambling, wins are typically marked by flashing lights and exciting jingles. These win-related sensory cues have been shown to increase physiological arousal responses to reward, as well as subjective ratings of enjoyment during gambling tasks (Cherkasova et al., 2018; Dixon et al., 2014; Loba et al., 2001). Recent studies have begun to investigate the role that win-related sensory cues play in decision-making. For instance, Cherkasova et al. (2018) found that participants were more likely to ignore probability information and choose high-risk gambles when rewards were accompanied by win-related sensory cues than when reward feedback was presented numerically without accompaniment. Similarly, Spetch et al. (2020) found that participants preferred to play—and recalled more high-reward payouts—on simulated slot machines with win-related sensory cues, than on equal-payout machines without such cues (see also Dixon et al., 2014).

While the above findings show that win-related sensory cues can bias overt choices, it remains to be determined where these cues exert their influence in the chain of information processing operations that lead to a choice being made. This study investigated whether win-related sensory cues influence one of the earliest steps in the decision-making process: attentional selection. Specifically, we investigated whether the addition of win-related sensory cues during post-trial feedback increases the likelihood that a reward-signalling stimulus will be prioritised by the attention system during a prior search task, relative to another reward-related stimulus that signals equivalent reward magnitude but with feedback that is not accompanied by win-related sensory cues. Participants completed a variant of the VMAC task in which the colour of a distractor circle signalled whether the reward on the current trial would have a bonus multiplier applied (*high-value distractor*), or not (*low-value distractor*). Another distractor type (*high-enriched distractor*) signalled the same bonus multiplier as the high-value distractor but was accompanied by win-related sensory cues during feedback. Critically, this design allowed us to assess whether the presentation of win-related sensory cues during feedback increased the attentional priority of the high-enriched distractor compared to the high-value distractor. Such a finding would demonstrate that win-related sensory cues exert an early influence on attentional prioritisation, shaping the process by which participants initially gather information about the options that are present and available, before any overt decision to choose one of these options is made. Furthermore, to verify the influence of win-related sensory cues on overt choice and memory for rewarding outcomes, participants were asked to choose which distractor type they would prefer to be presented with on a future trial and to estimate the average reward won on each trial type. In line with previous findings (Cherkasova et al., 2018; Dixon et al., 2014; Spetch et al., 2020), we predicted that participants would prefer the high-enriched distractor over the high-value distractor, and estimate higher average earnings on high-enriched distractor trials compared to high-value distractor trials.

## Method

### Participants and apparatus

Rusz et al. (2020) reported a meta-analytic standardized mean difference of 0.35 in studies of VMAC that use visual search paradigms. Previous studies examining the influence of win-related sensory cues on overt choice have demonstrated small to medium effect sizes (*d* = 0.23–0.70; Cherkasova et al., 2018; Spetch et al., 2020). Power analysis using G*Power (Faul et al., 2007) indicated that 66 participants would provide adequate power (~.80) to detect an anticipated within-subjects effect size of *d_z_* = 0.35. To account for anticipated dropouts and exclusions, we tested 75 UNSW Sydney students, of which 71 completed the study (43 females, 28 males; age *M* = 19.6 years, *SD* = 2.24). Participants received course credit for participation, and a 15 AUD supermarket voucher was available to participants whose final score was in the top quartile. Participants completed the experiment online in their web browser, with stimulus presentation controlled by jsPsych (de Leeuw, 2015). This study was approved by the UNSW Sydney Human Research Ethics Advisory Panel (Psychology).

### Stimuli and design

#### Visual search task

**Figure 1.**
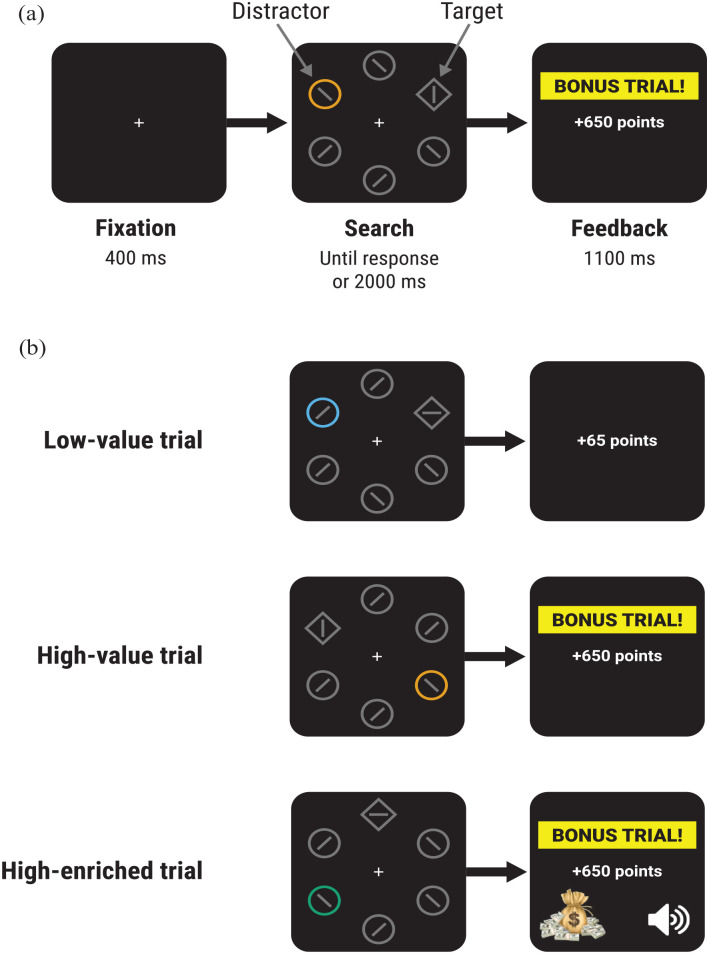
Experimental design: (a) Participants were initially presented with a fixation cross, followed by a search display. Participants were required to make a rapid keypress response to indicate the orientation of a line segment contained within the diamond-shaped target to earn point rewards according to their response speed. The colour of a colour-singleton distractor signalled the level of reward and type of feedback that would be delivered on that trial. (b) On low-value trials, participants received a base rate of reward and the feedback display showed only the number of points earned. On high-value trials, a 10× multiplier was applied to the reward, and feedback showed the number of points earned along with the text “BONUS TRIAL!.” On high-enriched trials, a 10× multiplier was applied to the reward, and feedback was the same as that on high-value trials with the addition of win-related sensory cues (a picture of a money bag and a “win” sound).

Each trial consisted of a fixation display, search display, and feedback display (see Figure 1a). Screen background was black. The fixation display consisted of a central white fixation cross. After 400 ms, the search display appeared, comprising five circle non-targets and a diamond target (80 × 80 pixels) arranged evenly around screen centre at an eccentricity of 140 pixels. Each circle contained a grey line segment randomly tilted 45° clockwise or anticlockwise from vertical. The diamond target contained a grey line segment randomly oriented horizontally or vertically. On most trials, one of the circles (termed the *distractor*) was coloured either blue (RGB: [37,141,165]), orange (RGB: [193,95,30]), or green (RGB: [54,165,65]); all other shapes were grey (RGB: [70,70,70]). The assignment of blue, orange, and green to the roles of high-value, low-value, and high-enriched colours (see below) was randomly determined for each participant. Distractor and target locations were determined randomly on each trial.

The participant’s task was to respond as rapidly and accurately as possible to the orientation of the line segment in the target, by pressing “C” (horizontal) or “M” (vertical). Correct responses earned points depending on response time (RT) and the colour of the distractor. For trials with a distractor in the low-value colour, or with no distractor in the display (*distractor-absent* trials), correct responses earned 0.05 points per ms that RT was below 1400 ms (e.g., an RT of 600 ms earned 40 points). Trials with a distractor in the high-value or high-enriched colour were “bonus trials,” with points multiplied by 10 (so an RT of 600 ms earned 400 points). Correct responses with RT above 1,400 ms or below 150 ms (anticipations) earned no points, and errors resulted in the loss of points that would otherwise have been won. The search display remained until a response was made or until a timeout after 2,000 ms. The feedback display then appeared for 1,100 ms. If the response was correct, feedback showed the number of points won (e.g., “+40 points”); if the response was incorrect, “ERROR” and the number of points lost was displayed (e.g., “ERROR: LOSE 40 points”). On high-value and high-enriched distractor trials, a yellow box labelled “BONUS TRIAL!” appeared above the reward feedback. On correct high-enriched distractor trials, reward feedback was accompanied by a win-related auditory cue (a casino-like, rising-pitch “win” sound) and visual cue (a cartoon picture of a bag of money, see Figure 1b); these cues were not presented on incorrect high-enriched distractor trials. If the trial timed-out, feedback stated “Too slow. 0 points.” If an anticipation was made, feedback stated “Please do not anticipate which response to make” and remained on-screen for an additional 1,400 ms to discourage anticipatory responding.

The search task comprised 16 blocks of 40 trials, for a total of 640 trials. Each block contained 10 trials with each distractor type (high-enriched, high-value, low-value) and 10 distractor-absent trials, in random order. The inter-trial interval was 1,000 ms.

#### Choice task

In each trial of the choice task, participants were presented with a circle rendered in one of the search-task distractor colours (orange, blue, or green) on the left and right of the screen. On each trial, participants were asked to choose (using the mouse, with no time pressure) which colour they would prefer to be in the display for the next search trial (although no such trial was subsequently presented), with instructions stating that the number of points associated with a rapid, correct response on a trial featuring that distractor would be added to their total score, such that they should pick the colour that would earn them more points. There were three different choice types: (1) high-enriched versus high-value; (2) high-enriched versus low-value; and (3) high-value versus low-value. Participants completed two trials of each choice type, in random order. The left/right position of the options was counterbalanced across the presentations of each choice type. After the participant made their choice, a feedback screen stated “We have added the points earned from this choice to your total” but did not reveal the specific number of points that was added.

#### Estimation task

In the estimation task, participants were shown a coloured circle and asked to estimate the average number of points that they had earned for a correct response when the search display contained a distractor in that colour during the search task. Participants provided one estimate for each of the three distractor colours in random order. Participants typed their estimate into a text box provided below an image of the circle. Responses were limited to integer values in the range 0 to 9,999.

### Procedure

To ensure that the audio on the participant’s computer was unmuted—thus enabling them to hear the auditory win cues presented on high-enriched trials—participants were required to enter a three-digit number that was recited by a recorded voice before starting the experiment. Participants were informed that their aim should be to earn as many points as possible, with the top-scoring 25% of participants receiving a supermarket voucher for 15 AUD. As further motivation, participants unlocked a new “medal” tier (bronze, silver, gold, platinum, diamond, or elite) for every 18,500 points earned. This value was chosen based on RTs from previous studies, such that the best-performing ~10% of participants achieved the “elite” medal tier.

Participants then received instructions for the search task, stating that (1) the faster a correct response was made, the more points would be earned; (2) when a circle was in the high-value or high-enriched colour, it would be a bonus trial and more points were available (participants were not instructed about the specific multiplier that was applied); and (3) that when a circle was in the low-value colour, it would not be a bonus trial. Importantly, the instructions emphasised that the participant’s task was to respond to the diamond and that the diamond would never be coloured, such that the best strategy in the search task was to ignore the coloured circles. Check-questions verified understanding of these instructions, with participants unable to continue until all responses were correct. Participants then completed the search task, taking a break after each block of trials, during which they were shown their total number of points, and an animation presented any medals that had been unlocked since the previous break. After completing the search task, participants completed the Choice task, followed by the Estimation task.

### Data analysis

For four participants, more than 15% of search trials were recorded as timeouts or anticipations; all data from these participants were excluded from subsequent analysis. After removing invalid responses, one additional participant had mean accuracy below 60%, and another participant had a mean RT more than 3.5 standard deviations above the sample mean; data from these participants were also excluded. For the remaining participants, we discarded data from the first two trials after each break, timeouts (0.2% of all trials), and anticipations (0.1% of all trials) as in previous protocols (e.g., Le Pelley et al., 2015; Pearson et al., 2016; Watson et al., 2019). Analysis of RTs used correct responses only. Primary analyses were conducted using *R* (version 4.0.3); the *afex* package (Singmann et al., 2022) was used for analyses of variance (ANOVAs), and the *lme4* package (Bates et al., 2015) was used for mixed effects models. Bayesian analyses were conducted using JASP (0.16.3.0; JASP Team, 2021). Greenhouse-Geisser-corrected degrees of freedom are reported where appropriate. Data, experiment, and analysis code are available at https://osf.io/upe8g/.

## Results

### VMAC task

**Figure 2.**
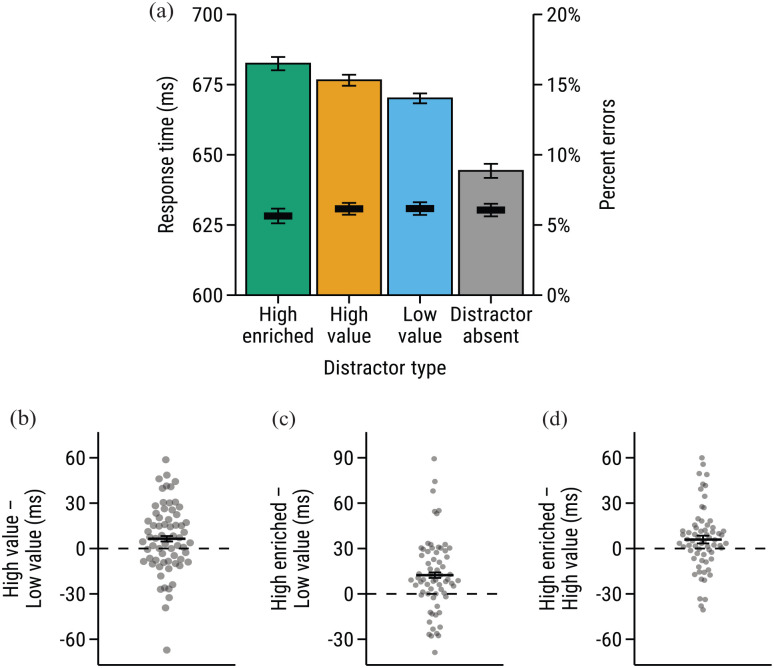
(a) Mean response times and percentage of errors for trials featuring each distractor type. Bars show the mean response time and superimposed black line segments show the mean percentage of errors. (b) VMAC effect. The VMAC effect is calculated as the response time on high-value distractor trials minus response time on low-value distractor trials. (c) Enriched VMAC effect. The enriched VMAC effect is calculated as the response time on high-enriched distractor trials minus response time on low-value distractor trials. (d) Enrichment effect. The enrichment effect is calculated as the response time on high-enriched distractor trials minus response time on high-value distractor trials. The black line segments in figures (b)-(d) show the mean effects. Individual subject means are displayed as grey points. Error bars in all figures represent within-subjects *SEM* (Morey, 2008).

Figure 2a shows RT and accuracy data across trials featuring each distractor type. RT data were first analysed via one-way repeated measures ANOVA, revealing a main effect of distractor type (high-enriched, high-value, low-value, distractor-absent), *F*(2.29, 148.56) = 60.1, *p* < .001, $\eta_{p}^{2}$ = .480. To further investigate this main effect, pairwise paired samples *t*-tests were conducted, with the Holm–Bonferroni method used to control the familywise error rate. These analyses confirmed that responses were slower on trials featuring a physically salient distractor compared to distractor-absent trials, suggesting that attention was captured by the physically salient distractors; high-enriched versus distractor-absent, *t*(65) = 9.80, *p* < .001, *Mdiff* = 38.20 ms, *d_z_* = 1.21; high-value versus distractor-absent, *t*(65) = 9.60, *p* < .001, *Mdiff* = 32.28 ms, *d_z_* = 1.18, low-value versus distractor-absent, *t*(65) = 10.18, *p* < .001, *Mdiff* = 25.83 ms, *d_z_* = 1.25.

Of more interest is the comparison between trials featuring a physically salient distractor (i.e., the high-enriched, high-value, and low-value distractor trials). The comparison between high-value and low-value distractor trials provides a measure of the effect of learned value on attentional capture, since each of these trial types featured a physically salient distractor, but differed in the magnitude of reward available. Planned *t*-tests confirmed that responses to the target were significantly slower when the search display contained a high-value distractor compared to a low-value distractor, *t*(65) = 2.31, *p* = .048, *Mdiff* = 6.45 ms, *d_z_* = 0.28. That is, participants showed a standard VMAC effect, suggesting that attention was more often captured by the high-value distractor than the low-value distractor (see Figure 2b). Responses to the target were also significantly slower when the display contained a high-enriched distractor compared to a low-value distractor, *t*(65) = 4.07, *p* < .001, *Mdiff* = 12.37 ms, *d_z_* = 0.50, indicating that participants also displayed a VMAC effect to the high-enriched distractor (Figure 2c). The critical contrast comes from the comparison of the high-enriched and high-value distractor trials, as both trial types featured a physically salient distractor that signalled a bonus, but differed in the type of feedback that was presented. Thus, the difference between these two trials types provides a measure of the influence of sensory win-cues on value-modulated attention. A planned *t*-test revealed that responses to the target were significantly slower on high-enriched trials than on high-value trials, *t*(65) = 2.31, *p* = .048, *Mdiff* = 5.92 ms, *d*_z_ = 0.28 (Figure 2d).

Error rates were low across all trial types. Analysis of errors using one-way ANOVA revealed a non-significant main effect of distractor type, *F*(2.83, 183.72) = 1.14, *p* = .331, $\eta_{p}^{2}$ = .017.

### Choice task

**Figure 3.**
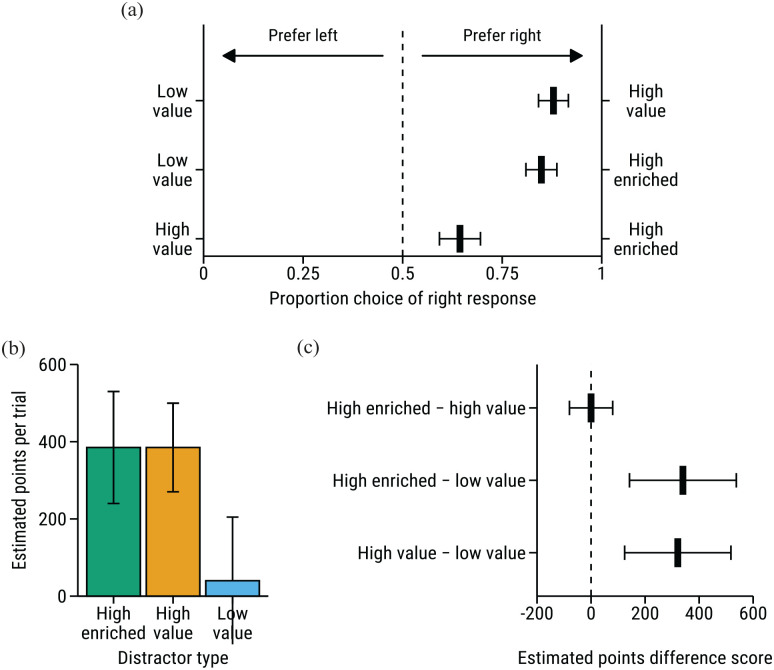
(a) Mean choice response for each choice type. Black line segments to the left of the dashed line indicate preference for the distractor type displayed on the left y-axis, line segments to the right of the dashed line indicate preference for the distractor type on the right y-axis. Error bars show *SEM.* (b) Median estimated points for each distractor type. (c) Median difference scores for pairwise comparisons of estimated points. Error bars for (b) and (c) show interquartile range (IQR).

Figure 3a shows the mean of the responses for each choice type. For each choice, selecting one of the options was coded as 0, and selecting the other option was coded as 1. We analysed choice responses for each of the three choice types using separate intercept-only mixed-effects logistic regression models, with random intercepts for participants. Wald *Z*-tests were used to determine whether the fixed-intercept parameter was significantly different from 0, which would indicate choice performance different from chance. As expected, participants showed a significant preference for both distractor types that were associated with high reward over the low-value distractor—high-value versus low-value, β = 9.92, *Z* = 5.26, *p* < .001; high-enriched versus low-value, β = 8.15, *Z* = 5.33, *p* < .001. The more interesting comparison comes from the high-enriched versus high-value choice: these distractor types signalled the same 10× reward multiplier but differed in whether feedback was accompanied by win-related sensory cues. Despite the equivalence in their associated reward magnitude, participants showed a significant preference for the high-enriched distractor over the high-value distractor, β = 1.18, *Z* = 2.36, *p* = .018.

### Estimation task

Figure 3b shows participants’ estimates of points earned on trials featuring each distractor type. Shapiro–Wilk’s tests indicated significant deviations from normality in the estimates for each distractor type (all *p*’s < .001). Wilcoxon signed-rank tests were therefore used to compare estimates (see Figure 3c). Relative to low-value distractor trials, participants estimated that they earned significantly more points on high-enriched distractor trials, *W* = 83, *Z* = 6.12, *p* < .001, and high-value distractor trials, *W* = 116.5, *Z* = 5.79, *p* < .001. One potential explanation for the influence of win-related sensory cues on attention is that their presentation inflated participants’ estimates of the number of points that they earned on trials containing the high-enriched distractor, relative to trials containing the high-value distractor. However, there was no significant difference between participants’ estimates for trials containing the high-enriched and high-value distractors, *W* = 783.5, *Z* = 0.55, *p* = .579. A Bayesian Wilcoxon signed-rank test was used to quantify the strength of the evidence in favour of this null result, which indicated moderate support for the null hypothesis, BF_01_ = 6.51.

## Discussion

Our findings provide clear evidence that win-related sensory cues enhance the attentional priority of reward-related stimuli. In line with previous findings (e.g., Le Pelley et al., 2015; Watson et al., 2019), participants displayed a VMAC effect to distractor stimuli that were associated with a high-value reward: responses to the target were slower (but no more accurate) when the search display contained either the high-value or the high-enriched distractor compared to the low-value distractor, even though this pattern of behaviour counterproductively resulted in reduced reward on trials in which higher magnitude rewards were available. The implication is that the attentional priorities of the high-value and high-enriched distractors were augmented as a consequence of their learned value, such that they became more likely to capture attention—and hence interfere with search for the target—than the low-value distractor. While it is well established that stimuli with high learned value are prioritised by the attention system, the key novel finding of this study is that responses to the target were slower when the search display contained the high-enriched distractor compared to the high-value distractor, indicating that the addition of win-related sensory cues to post-trial reward feedback further augmented the attentional priority of the high-enriched distractor.

Beyond their impact on attention, this study also demonstrated an influence of win-related sensory cues on explicit choice. When asked to select which distractor they would prefer to be presented with on an upcoming trial to maximise their reward, participants were more likely to choose the high-enriched distractor than the high-value distractor, even though the magnitude of the available reward signalled by each of these stimuli was equivalent. This finding is consistent with the idea that win-related sensory cues can bias overt choices (Cherkasova et al., 2018; Dixon et al., 2014; Spetch et al., 2020). Critically, the current findings go beyond this prior work by demonstrating that these cues influence the decision-making process from its earliest stages, where information is initially selected and prioritised by the attention system for further processing.

One explanation for this pattern of results is that the presentation of win-related sensory cues inflated the perceived value of the associated outcome, such that the point rewards delivered on high-enriched trials were perceived as having greater magnitude than the rewards on high-value trials. Evidence in line with this idea comes from studies showing that participants are more likely to recall high-value wins and recall winning more frequently on simulated slot machines that feature win-related sensory cues, than equivalent machines without such cues (Dixon et al., 2014; Spetch et al., 2020). Notably, in the current experiment, participants were not explicitly informed about the value of the bonus multiplier that was applied on high-enriched and high-value distractor trials. So, they may have interpreted the addition of win-related sensory cues on high-enriched trials as an indication that an especially large bonus multiplier was being applied on that trial, resulting in an objectively higher value reward than was available on high-value trials. However, in opposition to this idea, participants’ estimates of the average number of points earned on high-value and high-enriched trials were equivalent, suggesting that they had identified that the two trial types were associated with the same reward magnitude. Given that participants were significantly slower on high-enriched distractor trials than on high-value distractor trials, and RTs determined the magnitude of reward on each trial, one might argue that similar estimates for the high-enriched and high-value distractors indicates a bias in favour of the high-enriched distractor. However, there was no significant difference in the average reward received across these two trial types (see Supplementary Materials). Alternatively, the presentation of a positively valenced audiovisual cue during feedback may have provided a transient arousal signal that strengthened learning of the distractor colour-reward relationship on high-enriched trials. On this account, the win-related sensory cues increased participants’ engagement with the task during reward feedback, so they were better able to learn about the relationship between the high-enriched distractor and high-value reward. As a result of this facilitated learning, the high-enriched distractor would have a stronger association with high reward than would the high-value distractor, and so would be especially prioritised by attention. Evidence in line with the idea that win-related sensory cues can promote increases in arousal comes from Cherkasova et al.’s (2018) finding that the presentation of such cues increases pupil dilation, which can be used as a proxy measure of noradrenergic signalling in the locus coeruleus (LC). Theories of noradrenergic function suggest that high levels of phasic spiking in the LC (and thus changes in pupil size) are associated with task engagement and facilitate exploitation of the task being undertaken (Aston-Jones & Cohen, 2005). Future studies using pupillometry could help to shed light on whether arousal-induced strengthening of learning underlies the effect of win-related sensory cues on value-modulated attention observed here.

A third potential explanation is that the win-related sensory may have become reinforcing in their own right. Evidence from the conditioning literature suggests that otherwise-neutral stimuli can gain motivational significance through repeated pairing with reward, to the extent that their presentation can act as a reinforcer for other behaviour (i.e., *conditioned reinforcement*; Kelleher & Gollub, 1962; Williams, 1994). For example, when pigeons are trained that the delivery of a food reward to a magazine is immediately preceded by the sound of a food hopper being activated, they will subsequently learn to peck at a key to produce the sound of the food hopper alone, suggesting that the sound itself has become rewarding (Kelleher, 1961; Zimmerman et al., 1967). Extending this idea to the current findings, the co-occurrence of the win-related sensory cues and high-value rewards in the early stages of the task may have resulted in the cues acquiring reinforcing properties. Alternatively, or in addition, because the win-related sensory cues were only ever presented following correct responses, they may have come to be experienced as rewarding because of their association with the intrinsically rewarding experience of performing well in the task (Blain & Sharot, 2021). Finally, there is some evidence to suggest that any stimulus change associated with behaviour can be experienced as rewarding and so can motivate behaviour (e.g., Inglis et al., 1997; Osborne & Shelby, 1975).

Regardless of the specific mechanism that leads the win-related sensory cues to be experienced as rewarding in their own right, the result is that their presentation on high-enriched distractor trials may have been experienced as an additional degree of reward. Consequently, the high-enriched distractor would have a higher learned value than the high-value distractor (because the high-enriched distractor signalled both a high-value point reward *and* the rewarding sensory cues), thus resulting in increased attentional priority for the high-enriched distractor. This account would also explain why participants demonstrated a choice preference for the high-enriched distractor over the high-value distractor, while accurately identifying that the two trial types resulted in equivalent point rewards—the high-enriched distractor trials were experienced as more rewarding than the high-value trials despite signalling rewards of equivalent magnitude.

In summary, this study demonstrates that the presentation of win-related sensory cues can augment the attentional priority of reward-related stimuli. While previous findings have shown that win-related sensory cues modulate behavioural choices, particularly in the context of gambling tasks (Cherkasova et al., 2018; Dixon et al., 2014; Spetch et al., 2020), this study is the first to demonstrate that these cues exert an influence from the earliest stages of decision-making (i.e., attentional selection). These findings may have implications for our understanding of addiction-related attentional biases and their influence on behaviour, particularly in the context of gambling where win-related sensory cues are common.

## Supplemental Material

sj-docx-1-qjp-10.1177_17470218231160368 – Supplemental material for Value-modulated attentional capture is augmented by win-related sensory cues Click here for additional data file. Supplemental material, sj-docx-1-qjp-10.1177_17470218231160368 for Value-modulated attentional capture is augmented by win-related sensory cues by Daniel Pearson, Meihui Piao and Mike E Le Pelley in Quarterly Journal of Experimental Psychology
