# Supplementary material for: Value-modulated attentional capture is augmented by win-related sensory cues
Source: Q J Exp Psychol (Hove). 2023 Mar 23;77(1):133–43. doi: 10.1177/17470218231160368 (PMC10712205; doi:10.1177/17470218231160368)
Supplement: sj-docx-1-qjp-10.1177_17470218231160368 – Supplemental material for Value-modulated attentional capture is augmented by win-related sensory cues [file sj-docx-1-qjp-10.1177_17470218231160368.docx]

**SUPPLEMENTARY MATERIALS**

**TO ACCOMPANY**

Value-modulated attentional capture is augmented by win-related sensory cues

Daniel Pearson^1,2^

Meihui Piao^1^

Mike E Le Pelley^1^

1. School of Psychology, UNSW Sydney
2. School of Psychology, The University of Sydney

**Corresponding author:**

Dr Daniel Pearson

School of Psychology

The University of Sydney

NSW 2006

Australia

Email: [danielpearson90@gmail.com](mailto:danielpearson90@gmail.com)

**Analysis of average number of points earned for trials containing each distractor type**

The number of points that participants earned on each trial was a function of response time (faster responses meant that more points could be earned), the type of distractor that was present in the search display (a 10× bonus multiplier was applied to points earned on high-value and high-enriched distractor trials), and response accuracy (errors resulted in the loss of the points that would have been earned for a correct response).

Figure S1 shows the average number of points earned across all trials featuring each distractor type. A one-way repeated measures ANOVA revealed a main effect of distractor type (high-enriched, high-value, low-value, distractor-absent), *F*(1.24, 80.51) = 2288.1, *p* < .001, $\eta_{p}^{2}$= .972. Follow-up pairwise paired samples *t*-tests revealed that, as expected, participants earned significantly more points on trials in which a 10× bonus multiplier was applied to reward, than on trials in which no such multiplier was applied—high-enriched versus low-value, *t*(65) = 47.7, *p* < .001, *Mdiff* = 286.27, *d_z_* = 5.99; high-enriched versus distractor-absent, *t*(65) = 48.2, *p* < .001, *Mdiff* = 285.06, *d_z_* = 5.93; high-value versus low-value, *t*(65) = 50.2, *p* < .001, *Mdiff* = 285.27, *d_z_* = 6.18; high-value versus distractor-absent, *t*(65) = 49.8, *p* < .001, *Mdiff* = 284.06, *d_z_* = 6.13. Participants earned significantly fewer points, on average, on trials featuring a low-value distractor than on trials in which no physically salient distractor was present in the display, *t*(65) = 4.77, *p* < .001, *Mdiff* = 1.21, *d_z_* = 0.59. There was no significant difference in the average number of points earned on high-enriched and high-value distractor trials, *t*(65) = 0.36, *p* = .719, *Mdiff* = 1.00, *d_z_* = 0.04.


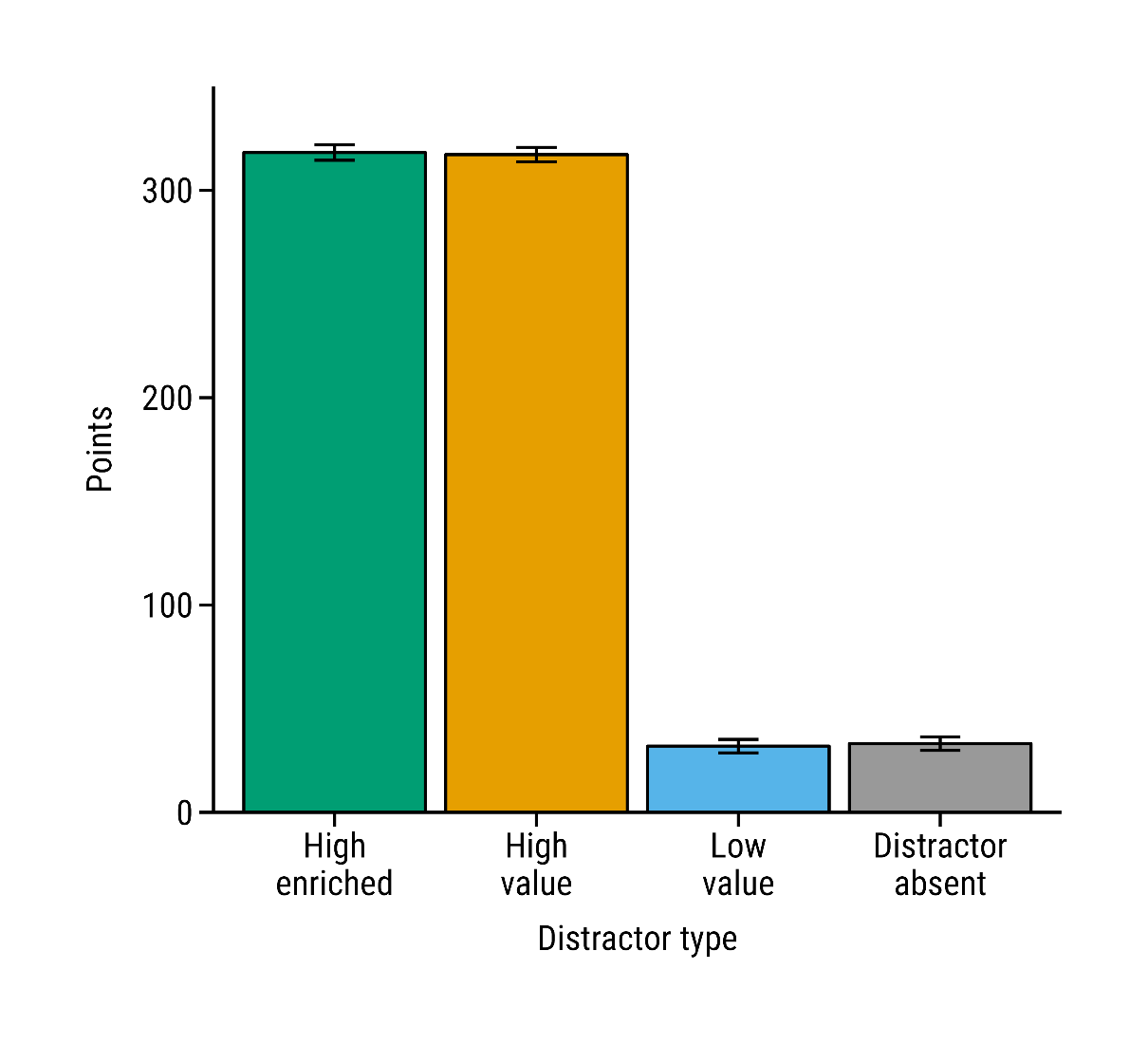


Figure S1. Mean points earned per trial for trials featuring each distractor type. Bars show the mean number of points earned. Error bars represent within-subjects SEM (Morey, 2008).
